# Factors affecting the cerebral network in brain tumor patients

**DOI:** 10.1007/s11060-012-0814-7

**Published:** 2012-02-14

**Authors:** Jan J. Heimans, Jaap C. Reijneveld

**Affiliations:** Department of Neurology, VU University Medical Center, De Boelelaan 1117, 1081 HV Amsterdam, The Netherlands

**Keywords:** Glioma, Neural networks, Cognition, Epilepsy

## Abstract

Brain functions, including cognitive functions, are frequently disturbed in brain tumor patients. These disturbances may result from the tumor itself, but also from the treatment directed against the tumor. Surgery, radiotherapy and chemotherapy all may affect cerebral functioning, both in a positive as well as in a negative way. Apart from the anti-tumor treatment, glioma patients often receive glucocorticoids and anti-epileptic drugs, which both also have influence on brain functioning. The effect of a brain tumor on cerebral functioning is often more global than should be expected on the basis of the local character of the disease, and this is thought to be a consequence of disturbance of the cerebral network as a whole. Any network, whether it be a neural, a social or an electronic network, can be described in parameters assessing the topological characteristics of that particular network. Repeated assessment of neural network characteristics in brain tumor patients during their disease course enables study of the dynamics of neural networks and provides more insight into the plasticity of the diseased brain. Functional MRI, electroencephalography and especially magnetoencephalography are used to measure brain function and the signals that are being registered with these techniques can be analyzed with respect to network characteristics such as “synchronization” and “clustering”. Evidence accumulates that loss of optimal neural network architecture negatively impacts complex cerebral functioning and also decreases the threshold to develop epileptic seizures. Future research should be focused on both plasticity of neural networks and the factors that have impact on that plasticity as well as the possible role of assessment of neural network characteristics in the determination of cerebral function during the disease course.

## Introduction

Optimal functioning of the brain depends on a large number of factors. Motor and sensory functioning, cognitive functioning, autonomic functioning, as well as emotional and social functioning all depend on anatomic and physiological integrity of the neural networks.

This integrity may be disturbed by numerous factors, varying from intrinsic brain disease to mood disorders and from metabolic disturbances and general disease to exogenous intoxications. Intrinsic brain disease may be focal, multifocal or generalized, and examples from these categories are brain tumors, multiple sclerosis and encephalitis, respectively. But optimal functioning of the brain is also determined by an intact environment. Cerebral functioning in a person who is generally ill as a result of infectious disease or disseminated cancer may be severely impaired. Consciousness and cognition may be hampered by a disturbed liver function, by hypoglycemia or by acidosis. And exogenous intoxications by carbon monoxide or alcohol, but also by numerous sorts of medication, such as sedative drugs, antidepressants and anti-epileptic drugs, may have a strong impact on functioning of the brain.

In patients with brain tumors, disorders of cerebral functioning are a major concern and it is important to realize that not only the tumor itself but also our broad array of therapeutic interventions and all kinds of metabolic, psychological and social factors contribute to the cerebral condition that determines how the patient is able to express his emotions, how he is able to think, how he functions in his work and in his social environment, and, finally, what his quality of life is.

### Brain tumors and cerebral function: general considerations

Brain tumors almost invariably cause severe symptoms, such as focal neurological deficits, cognitive deficits, and focal or generalized epileptic seizures. Apart from that, brain tumors may give rise to increased intracranial pressure, resulting in headache or impairment of consciousness. Brain tumors have this significant impact on the brain, since they force the non-tumoral brain tissue not only to adapt to the presence of an expanding mass, but also to the invasion of healthy brain tissue by infiltrating tumor cells. How this adaptive process takes place has yet to be elucidated. It is a great challenge to clarify the underlying mechanisms accounting for the changes in the brain’s functional status resulting from the presence of this foreign entity and leading to cognitive impairments, to an alteration of emotional functioning, and to epileptic seizures. When reviewing the current knowledge in neuro-oncology, there is a strong need for theory regarding the complex relations between the tumor on the one hand, and epilepsy and cognitive and emotional status on the other hand. The study of these relations is complicated by the fact that patients with primary brain tumors undergo a large number of therapeutic interventions, starting with surgery, which is almost invariably followed by irradiation, and in a number of cases by chemotherapy. Apart from these anti-tumor treatment modalities, brain tumor patients may receive antiepileptic drugs, antidepressants and corticosteroids, all having their specific impact on cerebral functioning.

### Brain tumors, cognition and epilepsy

Cognitive functioning may be impaired in many conditions that affect the brain. But “cognitive functioning” as such is an ill-defined entity. Cognition is a collective term for a number of functions that can be subdivided into different domains. The six most important domains are (1) information processing, (2) psychomotor functioning, (3) attention, (4) verbal memory, (5) working memory, and (6) executive functioning [1]. In order to get properly informed on the cognitive abilities of an individual patient, a number of cognitive tests should be performed, measuring these separate functions. The more extensive the test battery, the more detailed the information on the cerebral functioning of the patient.

A number of studies on cognitive functioning in glioma patients have been published. Most brain tumor patients experience cognitive deficits at some point during their disease. Severe neuropsychological impairments have been found in up to 89% of patients with high-grade gliomas (HGG) [2–4]. A comparable percentage of patients with low-grade gliomas (LGG) display cognitive deficits [5–12]. The most striking collective finding of these studies is that cognitive deficits are not restricted to the area of the brain where the tumor is located and where it has caused local damage, but rather should be traced to a more global dysfunctioning of the brain: memory disturbances, loss of concentration, difficulties with planning and language, and psychomotor slowness.

It appears that the rate of tumor growth is related to the degree of cognitive dysfunction: a fast-growing brain tumor causes more profound cognitive deficits than a slow-growing tumour [13, 14]. This is in accordance with the observation that brain tumor patients show remarkable preservation of cognitive functioning when compared to patients with acute lesions of the same size [15]. When comparing the devastating effect of these acute lesions with that of slowly growing tumors, it is clear that plasticity plays a major role in the resilience to cognitive deficits in brain tumor patients.

Epilepsy could be considered as one of the manifestations of brain dysfunction and is often the first manifestation of a brain tumor. Between 10 and 15% of adult patients presenting with epileptic seizures are diagnosed with a brain tumor as the underlying cause of the seizures [16, 17]. Conversely, most patients suffering from brain tumors develop epileptic seizures at some point during the course of their disease. This is the case in 85% of LGG patients, whereas about 50% of HGG patients will experience epileptic seizures at some point of their disease [18]. We do not know why some patients develop epileptic seizures whereas others, suffering from the same tumor type in the same location, do not.

### Specific impact of the different treatment modalities

Surgery, radiotherapy, anti-epileptic drugs, glucocorticoids and various chemotherapy regimens may have influence on cerebral functioning of brain tumor patients. But also the tumor itself, psychological distress, depression and fatigue have a certain impact, and usually a combination of all these factors eventually destines how the patient will function. This makes it difficult to sort out what the contribution of separate treatment modalities may be.

The primary aim of surgery is to reduce tumor mass if the localization of the tumor allows this. Talacchi and coworkers (2011) reported a series of 29 glioma patients in whom extensive neuropsychological examination was performed both pre- and postoperatively. They found postoperative impairment in comparison to preoperative functioning in 38% of patients and the reverse (postoperative improvement in comparison to preoperative functioning) in 24% of patients [19]. This illustrates that surgical treatment of brain tumors may be of benefit to cognitive function, since it reduces intracranial pressure and compression of brain tissue, but may also cause damage whether transient or more permanent.

Another study in 23 patients harboring a LGG in the language areas stressed the importance of neuropsychological assessment before operation: the authors found transient worsening of verbal working memory immediately after surgery which recovered within three months [20].

Improvement of language function after initial worsening following tumor resection was also reported by Sanai et al. [21].

A lot has been written on the role of radiotherapy on cerebral functioning. Especially the first reports on cognitive damage in young children who had been treated with radiotherapy for brain tumors or acute leukemia were alarming. Later, radiation induced neurotoxicity was also reported in adults who had been treated with radiotherapy for primary brain tumors or brain metastases [22, 23].

We performed a study on cognitive functioning among 195 patients with LGG [1]. Our findings suggested that a mean of 6 years after diagnosis the tumor itself, rather than the radiotherapy had the most deleterious effect on cognitive functioning; only high fraction dose radiotherapy (> 2 Gy) resulted in significant added cognitive deterioration.

In a follow-up study of the same cohort at a mean of 12 years after first diagnosis, however, it appeared that long-term survivors of LGG who did not have radiotherapy had stable cognitive status in contrast to patients who had received radiotherapy [24]. The latter group showed a progressive decline in attentional functioning. It is important to realize that this attentional deficit developed independently of the localization of the tumor and that treatment in this patient group had been restricted to focal irradiation. This finding indicates that global cognitive dysfunctioning may result from local damage.

In patients with HGG, it is more difficult to determine the long term role of radiotherapy because in these patients the duration of survival is much shorter than in LGG patients. Similar to surgical treatment, radiotherapy in HGG often leads to tumor response and this may result in improvement of cognitive functioning [25]. A rapid decline of cognitive functioning occurs in almost all cases of glioma when tumor progression or tumor recurrence occurs.

Glioma patients usually receive various kinds of drugs during the course of the disease. Epileptic seizures necessitate the prescription of anti-epileptic drugs, and especially the older or first-generation anti-epileptic drugs (phenytoin, carbamazepine and valproic acid) may have a negative influence on cognition. It is interesting and important to note that levetiracetam, a second generation drug, may have a positive influence, instead of a deteriorating influence on cognition [26].

Cerebral vasogenic edema may accompany glioma progression and cause neurologic deterioration. Glucocorticoids are usually very effective in the treatment of vasogenic edema, but may also induce new problems with regard to cognitive and emotional functioning [27].

Nowadays, temozolomide is incorporated in the treatment of most newly diagnosed HGG’s. The use of temozolomide is not associated with neurocognitive side effects [28] and may even have a beneficial effect on seizure frequency [29], but it is very well possible that other drugs will become part of glioma combined treatment regimens in the future and some of these drugs may prove to have neurotoxic side effects.

In conclusion, brain tumors may negatively affect cerebral functioning and various combinations of all therapeutic modalities, as summarized here, may have an additional negative, but in some situations also a positive impact. The study of brain functions and the way these functions are affected by tumor progression and by various treatment modalities is usually performed by means of measurement of function: neurological examination, a variety of neuropsychological function tests, performance status scales and quality of life questionnaires are examples of instruments to—directly or indirectly—assess brain function. All these functions, however, depend on the integrity of the cerebral network and direct measurement of network parameters would have obvious advantages. Electroencephalography (EEG) and magnetoencephalography (MEG) are potential instruments to study these parameters. If it would become possible to identify certain characteristics of the human cerebral network, more insight into the effects of local lesions, such as brain tumors, and into the effects of systemic treatments on the integrity and plasticity of the cerebral network could result. We will give a short overview of network theory and the possibilities to study neural networks in the following paragraphs.

### Network theory and the application in brain research

Network or graph theory originates from both the fields of mathematics and sociology. Combining these two has led to a number of methods of analyzing different types of networks by representing them in an abstract, theoretical figure called a ‘graph’. The challenge of the study of networks—including the study of neural networks—is to find the optimal method of describing and defining all kinds of biological and social networks. Networks usually combine two seemingly opposing concepts: integration and segregation. Most optimal functioning networks have so-called ‘small-world’ characteristics, referring to a locally clustered architecture in combination with long distance connections. This means that parts of the network that are virtually remote from each other can actually be linked through only a few steps. This co-existence of integration and segregation in complex networks has only recently been elucidated.

**Fig. 1:**
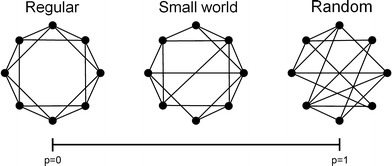
Schematic drawing of a random, a regular, and a small-world network. In the regular network (*left*), each node is only connected to its neighbors. Therefore, it has both a high clustering coefficient (C) and a long path length (L), while the random network (*right*) combines a low C and a low L. The intermediate of the two: the so-called “small-world network” (*middle*) can be achieved by relocating but a few long-distance connections from the regular network, which causes L to decrease drastically but preserves a high C. Thus it combines “the best of two worlds”

Random networks were first described in the 20th century and seemed promising to model complex networks [30, 31]. However, these graphs did not meet up to the expectations of explaining the abovementioned small-world characteristics of networks. Watts and Strogatz [32] provided an elegant way of modeling small-world networks. They proposed a very simple model of a one-dimensional network (see Fig. 1). In the “regular” network, each node or vertex is only connected to its ‘k’ nearest neighbors (k being the degree of the network). Next, with likelihood ‘p’, connections or edges are chosen at random and connected to other nodes, also chosen randomly. With increasing p, more and more edges become randomly reconnected and finally, for p = 1, the network is completely random. This comprehensible model allows investigation of all types of networks, ranging from completely regular to completely random.

The intermediate between random and regular architecture proved to be crucial to the understanding of the small-world phenomenon. Two measures appear to be important for the classification of a network on the continuum of regular to random. The first measure is the “clustering coefficient” (C), which can be defined as “the likelihood that neighbors of a vertex are also connected”. The second measure is the so-called “path length” (L). This is defined as “the average of the shortest distance between pairs of nodes counted in the number of edges”. Using these two measures, we can resume the theory as follows: regular networks are very clustered (high C) but it takes a lot of steps to get from one side of the graph to the other (high L). In contrast, random networks lack this high clustering (they have a low C) and the path length is short (low L). Neither of the two have small-world properties. However, these small-world properties occur already when p is only slightly higher than 0: now the path length L drops sharply, while the clustering coefficient (C) hardly changes. Thus networks with only a few (randomly) rewired connections combine both high clustering and a small path length: this phenomenon is referred to as the small-world phenomenon. These measures C and L make it possible to define the index of “small-worldness” [33].

The discovery of these tools to describe small-world networks initiated a widespread interest in all kinds of complex networks and gave rise to a wide range of theoretical and experimental studies. One of the most intriguing, and certainly the most complex network is the human brain. Brain activity is commonly studied by making use of functional magnetic resonance imaging (fMRI), EEG or MEG. EEG has been a routine instrument in the study of brain function for a number of decades. It measures electrical flow and is especially useful for the diagnosis of epilepsy and other pathological cerebral conditions, such as encephalitis. MEG is a more sophisticated method to measure brain activity and has a higher spatial resolution. MEG registers brain activity by measuring magnetic flow, and the scalp and the skull do not distort signal registration in contrast with EEG. Within the signal measured by EEG and MEG, different frequency bands can be distinguished: delta (0.5–4 Hz), theta (4–8 Hz), lower alpha (8–10 Hz), upper alpha (10–13 Hz), beta (13–30 Hz), lower gamma (30–55 Hz), and upper gamma band (55–80 Hz).

**Fig. 2:**
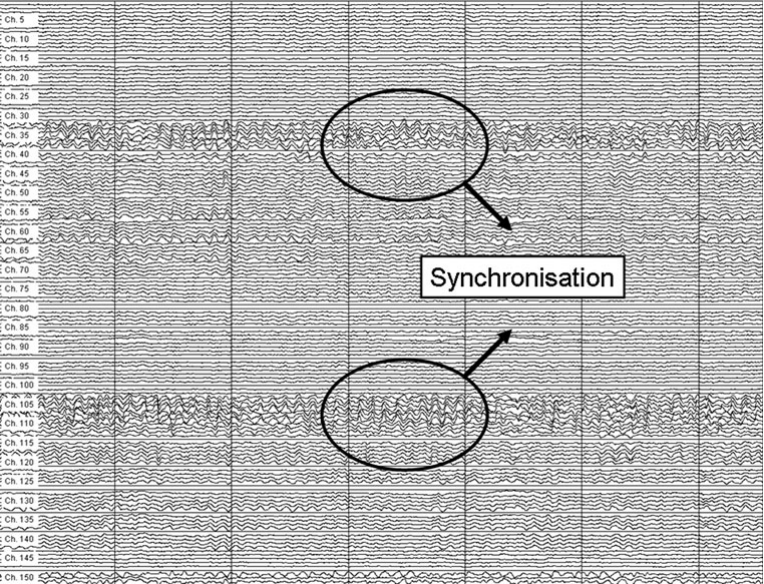
Part of a 151-channel MEG recording. Synchronization of signals from different regions of the brain is a measure of connectivity of these regions

A fundamental conception for the study of brain dynamics is “synchronization” or “functional connectivity”. The basic assumption of functional connectivity is that statistical interdependencies between time series of brain activity at separate areas reflect functional interactions between these brain regions [34]. This functional connectivity can be calculated on the basis of the amount of synchrony of brain activity measured in two different areas. The conception is that multiple local networks are maintained by long-distance patterns of functional connectivity and this results in higher and complex brain functions, such as planning, memory, and executive functioning [35–38]. Functional connectivity between brain areas may thus be used to construct graphs of the brain (see Fig. 2 for an example in an MEG recording). The two prerequisites of local segregation, referring to local specialization in specific tasks, and integration, combining information from lower-level networks at a higher and more global level, are thought to be crucial for optimal brain functioning [39–41]. The small-world network is a highly adequate model of organization in the brain, because it supports both segregated as well as integrated information processing [42]. Brain tumors may interfere with neuronal structures and the resulting disturbances of anatomical connectivity may lead to alterations of functional connectivity patterns [43, 44].

### Functional connectivity and neural networks in the presence of a brain tumor: the impact on cognition and the impact on epilepsy

During the last decade, we have shown that most LGG and HGG patients perform significantly worse than healthy controls on almost all cognitive tests. In order to better understand why glioma patients develop those global cognitive deficits, we investigated the correlation between resting-state MEG-based functional connectivity and cognitive functioning in patients with LGG [45]. We therefore studied correlations between time series of 126 MEG channels and we used the so-called “synchronization likelihood” (SL) as one of the measures of synchronization (or functional connectivity). The SL varies between zero (no synchronization at all) and one (total synchronization).

We found that LGG patients showed increased SL in the delta, theta, and lower gamma frequency bands in comparison to healthy controls, while connectivity in the lower alpha band was decreased. In another study we also found a correlation between cognitive functioning and network architecture in LGG patients [46].

These findings partly confirmed our previous studies, in which we also found changes in functional connectivity and network architecture in brain tumor patients with various histologies [47, 48]. There were some contradictory details between the earlier publications and the results of the later papers, especially regarding differences between tumor patients and healthy controls in the pattern and the direction of changes in connectivity in the various frequency bands. Possible explanations for these differences in both functional connectivity and network topology may relate to the different patient samples: a homogeneous group of LGG patients versus a more heterogeneous sample of brain tumor patients.

Despite these contradictory details, it is most likely that conceptions like functional connectivity and network topology are pivotal for cognitive functioning. Both cognitive performance and functional connectivity are disturbed in LGG patients, and correlations exist between these two phenomena. Brain tumor patients consistently show pathologically increased connectivity particularly in the lower frequency bands (delta, theta), which is related to poorer cognitive functioning.

We further investigated network characteristics in glioma patients with epilepsy by means of MEG registrations [49]. Functional connectivity was calculated in six frequency bands, as were a number of network measures such as normalized clustering coefficient and path length. Increased theta band connectivity appeared to be related to a higher total number of seizures. Furthermore, higher number of seizures was related to a less optimal, more random brain network topology. Other factors were not significantly related to functional connectivity or network topology. These results indicate that (pathologically) increased theta band connectivity is related to a higher number of epileptic seizures in brain tumor patients, suggesting that theta band connectivity changes are a hallmark of tumor-related epilepsy. Furthermore, a more random brain network topology is related to greater vulnerability to seizures. Thus, functional connectivity and brain network architecture may prove to be important parameters of tumor-related epilepsy.

## Concluding remarks

Brain tumors and brain tumor treatment lead to disturbances in cerebral function. These disturbances may consist of function loss (especially loss of complex functions), and epileptic seizures. It has been shown that both types of cerebral function disruption are associated with changes in functional connectivity and network architecture. Although we are only in the very beginning of unraveling the extremely complex network architecture of the brain, the findings in brain tumor patients may prove to be of great importance for our future strategies in the treatment of these patients. Possibilities to successfully combine brain tumor surgery and epilepsy surgery will improve, and we will be able to longitudinally study the influence of various treatment strategies on the network. In short, we will be able to study the plasticity of the brain in a direct and non-invasive way.
